# Awareness, Risk Perception, and Protective Behaviors for Extreme Heat and Climate Change in New York City

**DOI:** 10.3390/ijerph15071433

**Published:** 2018-07-07

**Authors:** Jaime Madrigano, Kathryn Lane, Nada Petrovic, Munerah Ahmed, Micheline Blum, Thomas Matte

**Affiliations:** 1 RAND Corporation, 1200 South Hayes Street, Arlington, VA 22202, USA; 2 Bureau of Environmental Surveillance and Policy, New York City Department of Health and Mental Hygiene, New York, NY 10013, USA; klane1@health.nyc.gov (K.L.); mahmed4@health.nyc.gov (M.A.); 3 The Earth Institute, Columbia University, 2910 Broadway, New York, NY 10027, USA; nada.petrovic@gmail.com; 4 Marxe School of Public and International Affairs, Baruch College Survey Research, One Bernard Baruch Way, New York, NY 10010, USA; micheline.blum@baruch.cuny.edu; 5 Vital Strategies, 61 Broadway, New York, NY 10006, USA; tmatte@vitalstrategies.org

**Keywords:** extreme heat, climate change, vulnerable populations, risk perception, public health preparedness

## Abstract

Preventing heat-related illness and death requires an understanding of who is at risk and why, and options for intervention. We sought to understand the drivers of socioeconomic disparities in heat-related vulnerability in New York City (NYC), the perceived risk of heat exposure and climate change, and barriers to protective behaviors. A random digit dial telephone survey of 801 NYC adults aged 18 and older was conducted from 22 September–1 October, 2015. Thirteen percent of the population did not possess an air conditioner (AC), and another 15% used AC never/infrequently. In adjusted models, odds of not possessing AC were greater for non-Hispanic blacks compared with other races/ethnicities, odds ratio (OR) = 2.0 (95% CI: 1.1, 3.5), and for those with low annual household income, OR = 3.1 (95% CI: 1.8, 5.5). Only 12% reported going to a public place with AC if they could not keep cool at home. While low-income individuals were less likely to be aware of heat warnings, they were more likely to be concerned that heat could make them ill and that climate change would affect their health than participants with a higher household income, OR = 1.6 (95% CI: 1.0, 2.3). In NYC, lack of access to AC partially explains disparities in heat-related health outcomes. Our results point to opportunities for knowledge building and engagement on heat-health awareness and climate change adaptation that can be applied in NYC and other metropolitan areas to improve and target public health prevention efforts.

## 1. Introduction

Extreme heat is a global health threat and one of the leading weather-related causes of death in the United States (US) [1]. Unusually hot summer days and nights have become more common over the last few decades, and heat waves are expected to become more frequent and severe with continued climate change [2]. Improving public health interventions that address extreme heat requires an understanding of who is at risk, the reasons for this heightened susceptibility, and plausible options for intervention.

An extensive literature on vulnerability to heat-related morbidity and mortality has been reviewed elsewhere [3,4]. Areas with lower prevalence of air conditioning (AC), for example, have been found to have higher rates of heat-related deaths [5,6], while areas with more green space tend to have lower rates of heat-related deaths [7,8]. At the individual level, older adults, men, and people with chronic physical and mental health issues have greater risk of heat-related morbidity and mortality [9]. Additionally, in many studies in the US, black individuals have been found to be more vulnerable to heat-related morbidity and mortality [3]. This is also true in New York City (NYC), where heat wave deaths were more likely to occur among non-Hispanic black individuals than other race/ethnicities [8]. However, less is known about why this population is at heightened risk. 

Non-Hispanic black individuals may be more vulnerable because they are more likely than non-Hispanic whites to live in areas lacking tree canopy and covered in impervious surfaces [10]. Race/ethnicity is also often associated with other socioeconomic disadvantages [11], which generally contribute to worse outcomes across a number of health conditions and may also limit access to air conditioning (AC). Institutionalized racism continues to perpetuate differences in health outcomes [12] as a result of oppression of blacks in all classes, independent of income [13]. Disentangling these complex factors to explain observed racial disparities in heat-related illness and death is critical for protecting vulnerable populations.

Awareness of heat warnings and risk perception may also play a role in disparities in heat-related vulnerability [14]. The extent to which individuals perceive risks associated with heat waves, which may be influenced by sociodemographic factors [15], can determine preventative actions they choose to take [16]. Moreover, risk perception of local natural hazards may inform perception of larger, more abstract topics, such as climate change risk [17]. A deeper understanding of how closely people link climate change to threats to their personal health and safety can improve climate change communication strategies and promote adaptive behaviors to protect health [18,19].

As in other metropolitan areas, NYC implements recommended best practices through a coordinated multi-agency approach during heat emergencies, described elsewhere [14]. Individuals at high health risk are advised to visit a cooling center or use AC set at 78 °F or “low cool” to lessen strain on the energy grid while protecting health. The NYC Department of Health and Mental Hygiene (DOHMH) promotes the Home Energy Assistance Program (HEAP) [20], which provides limited funding for low income individuals with medical needs to use towards the purchase and installation of ACs. The DOHMH also conducts outreach during the spring and summer to medical and service providers to provide guidance on how to help vulnerable patients and clients protect themselves during hot weather.

To better understand reasons for socioeconomic disparities in heat-related vulnerability and inform public health protection efforts, we examined heat-health awareness, perceived risk, and protective behaviors by race/ethnicity and income by repeating and expanding a telephone survey of NYC adults, originally conducted in 2011 in NYC [14].

## 2. Materials and Methods

A random digit dial telephone survey of 801 New York City adults aged 18 and older was conducted under the direction of Baruch College Survey Research from 22 September–1 October, 2015. The landline sample was based on a random digit dial (RDD) design which draws numbers from all existing landline telephone exchanges in the five boroughs of NYC, giving all phone numbers, listed and unlisted, a proportionate chance of being included. Respondents in the landline sample were selected randomly within the household. The cell phone sample was randomly selected using a wireless/mobile number database containing all possible numbers in 100-blocks dedicated to wireless service and 100-blocks providing shared services with no directory-listed telephone numbers in the five NYC boroughs. Cell phone respondents were screened for age and NYC residence. The data were weighted to the US 2010 Census population data for NYC for age, sex, race, Hispanic origin and borough. The estimated average sample tolerance for data from the survey is ±3.5 percentage points for the full sample of 801. Error for subgroups is higher. 

The 17-question survey was timed to take place at the end of the heat season. The questions, asked in English or Spanish, were closed ended, with the exception of a free response option for a question about stay-at-home behavior during very hot weather. As in the 2011 survey, questions pertained to AC ownership and use, awareness of heat warnings and hot weather behavior, and respondent age, gender, race/ethnicity, and household income. New questions on the perceptions of the dangers of heat and climate change were included, as was the free response for the stay-at-home behavior question. Adults reporting that they thought it was “somewhat unlikely” or “very unlikely” that heat inside their homes could cause them to become ill were defined as a “low risk perception” group. Adults reporting that they were “somewhat concerned” or “very concerned” that more frequent heat waves due to climate change will be bad for your health were defined as a “concerned” group. Lacking access to AC was defined as not having a functioning AC. Low-income households were defined as those reporting a household income of less than $30,000 per year, consistent with the analysis of our 2011 survey. The data were examined through the calculation of frequencies, proportions, measures of dispersion, and logistic regression models, conducted using SAS version 9.4 (SAS Institute, Cary, NC, USA) surveyfreq and surveylogistic procedures. This research was reviewed by the NYC DOHMH Institutional Review Board and was determined to be exempt. 

## 3. Results

The survey cooperation rate defined according to the American Association for Public Opinion Research Cooperation Rate 3 criteria was 51%, and the overall Response Rate 3 was 22% (The American Association for Public Opinion Research. Standard Definitions: Final Dispositions of Case Codes and Outcome Rates for Surveys. 7th edition. AAPOR. 2011. The contact rate for the present survey was 56%. http://www.aapor.org/AM/Template.cfm?Section=Standard_Definitions2&Template=/CM/ContentDisplay.cfm&ContentID=3156). Of the interviews, 90% (*n* = 717) were conducted in English and 10% (*n* = 84) in Spanish. Landline interviews accounted for 70% (*n* = 560) of all interviews; all others were cell phone interviews (*n* = 241). Table 1 displays the descriptive characteristics of survey respondents. 

When asked about the existence of a functioning AC anywhere in their home, 13% (95% CI: 10, 15) of the population responded that they did not have one. Among those who did not own AC, the most common explanation, given by 40% (95% CI: 30, 50), of participants, was inability to afford it (Table 2). This was followed by a response of “don’t need it” (33%, 95% CI: 23, 42). Although the majority of the population owned AC, 15% (95% CI: 12, 18) stated that they used it “less than half the time” in the preceding summer during very hot weather. The top two reasons that AC owners curbed their use were their electricity bill and a desire to conserve electricity, 24% (95% CI: 18, 30) and 21% (95% CI: 16, 27), respectively (Table 2).

When asked what they do when they cannot keep cool at home, the participants most frequently responded that they “stay at home even though hot” (47%, 95% CI: 41, 53). Only 12% (95% CI: 8, 16) of this group reported going to a public place with AC, such as a community center or library. Among respondents who chose not to leave their home, three in ten reported that they do not leave their home to find a cooler place because they “don’t think heat is dangerous” (30%, 95% CI: 22, 38) and more than a quarter volunteered that this was their personal preference (28%, 95% CI: 21, 36). A smaller subset expressed a wish to avoid spending time with strangers (12%, 95% CI: 7, 18). A majority of the population reported checking in on a family member, friend, or neighbor to make sure that they were okay during very hot weather (59%, 95% CI: 55, 62). 

Race/ethnicity and household income were strongly associated with AC access (Table 3). Non-Hispanic blacks were twice as likely to not have AC, compared with the rest of the population, (OR = 2.0, 95% CI: 1.1, 3.5), after adjusting for household income, gender, age, and low risk perception of the dangers of heat. Similarly, the odds of not having AC were three times greater for those with a household income less than $30,000 per year compared to those with greater household incomes (OR = 3.1, 95% CI: 1.8, 5.5) after adjustment for race, gender, age, and risk perception. In the sample of participants who possessed functioning AC, demographic or perception factors were not significantly associated with AC use.

We also examined heat-health awareness and behaviors, risk perception, and climate change concern among New Yorkers. A majority of participants reported seeing, hearing, or reading warnings about dangerously hot weather in NYC during the preceding summer (66%, 95% CI: 63, 70), but only 43% (95% CI: 39, 46) reported a belief that on very hot days, the heat inside their home is very likely or somewhat likely to make them ill. In a logistic regression model of race, income, gender, age, and risk perception predicting warning awareness, survey participants with a household income less than $30,000 were almost half as likely to report seeing, hearing, or reading heat warnings in NYC compared to individuals with higher household incomes (OR = 0.6, 95% CI: 0.4, 1.0) (Table 4). Low risk perception was also strongly associated with lower warning awareness (OR = 0.6, 95% CI: 0.4, 0.9). We found that participants with a low household income (OR = 2.0, 95% CI: 1.3, 3.0), non-Hispanic blacks (OR = 1.9, 95% CI: 1.2, 3.0), and participants who had seen, heard, or read about heat warnings (OR = 1.7, 95% CI: 1.2, 2.6) were more likely to check in on a family member, friend, or neighbor to make sure they were okay during very hot weather. 

In a logistic regression model examining risk perception, people with low household incomes were nearly twice as likely to believe that, on very hot days, the heat inside their home is very likely or somewhat likely to cause them to become ill, controlling for race, household income, gender and age (OR = 1.9 (95% CI: 1.3, 2.8) (Table 4). Males were less likely to hold this belief than females, OR = 0.6 (95% CI: 0.4, 0.9). Similarly, participants age 65 and older had a lower odds of holding this belief, compared to those under the age of 65, OR = 0.7 (95% CI: 0.5, 1.1), although the association was not statistically significant (*p* < 0.1).

Finally, survey participants were asked about their concern for heat waves within the context of climate change. Overall, 68% (95% CI: 64, 71) reported being “very concerned” or “somewhat concerned” that more frequent heat waves due to climate change would negatively impact their health. Participants with a household income lower than $30,000 were more likely to express this concern than participants with a higher household income (OR = 1.6, 95% CI: 1.0, 2.3) and older individuals were somewhat less likely to be concerned than younger individuals (OR = 0.7, 95% CI: 0.5, 1.0) (Table 4).

## 4. Discussion

### 4.1. Racial and Economic Disparities in AC Access

We found that 28% of New Yorkers did not have access to a functioning AC or used it less than half the time or not at all during very hot weather. These findings were similar to the 2011 survey performed by the DOHMH, which found that 11% of New Yorkers did not have access to a functioning AC and 14% used it less than half the time or not at all during very hot weather [14]. Our findings indicate that racial and socioeconomic disparities in heat illness and death are partially explained by AC access. Non-Hispanic blacks and individuals with a household income of less than $30,000 per year, two groups found in studies to be more vulnerable to heat [8], were both independently less likely to possess AC than those of other races/ethnicities and those with higher household incomes. The most frequently reported reasons for lack of AC or lack of AC use were financial barriers and additionally, for AC use, a desire to conserve energy (which may also indicate a financial barrier). The next most frequent reason for not owning an AC was reportedly not needing it, indicating an opportunity for knowledge building on the serious health consequences of heat exposure. 

Notably, non-Hispanic blacks were less likely to own an AC even when adjusting for household income, suggesting that lack of financial resources is not the only cause of lower AC ownership among this group. Similar disparities for black Americans that cannot be fully explained by income are also seen for a variety of health outcomes (including early death rates, diabetes and obesity, hypertension) [21], as well as economic and educational outcomes [22]. The results may be indicative of the “pernicious effects of race-based discrimination” [21], which limits access to resources and services, as well as opportunities for social mobility [23]. Traditional measures of SES are not equivalent across race because, for example, compared to whites, blacks may have less purchasing power because the costs of goods and services are higher in black communities [24]. Traditionally disadvantaged groups may be more likely to live in buildings that do not have wiring conducive to AC installation, although building wiring was not cited as a main reason for not owning AC in the 2011 survey among all New Yorkers. There may be other explanations for how economic and structural racism impact AC access. Further qualitative research is needed to better understand these disparities and sharpen interventions to address root causes [25]. It is also important to note that other factors, including cultural/religious [26] and occupational [27], have also been shown to contribute to heat-related vulnerability.

These results also point to the continued need to expand AC access among vulnerable low-income and non-Hispanic black individuals. To offset increased AC use in heat emergency situations, when the electric grid can be stressed, strategies are needed to promote more equitable use of AC, including reduced commercial use. Ensuring that those who need AC to protect their health have access to it, while also being mindful of energy conservation issues, can help reduce electric grid strain during extreme heat events, while protecting the health of the most vulnerable. Further, research integrating building design, thermal comfort, and climate change adaptation is needed [28].

### 4.2. Perceptions of Heat and Climate Change Risk

Heat warning systems are meant to alert the public to the dangers of heat and enable protective actions to be taken. In NYC, the activation of emergency response plans, including public messaging, are triggered when the maximum heat index is forecast to reach 100 °F for any period of time, or 95 °F for at least two consecutive days [14]. During the summer of 2015, approximately two-thirds of New Yorkers had seen, heard, or read a heat warning, fewer than the 79% observed in 2011 [14]. Recall bias may be a possible reason for this; the 2011 survey followed a season with severe heat, which peaked at 115 °F, while the 2015 heat season was more moderate, with four days meeting heat emergency criteria, as opposed to six in 2011. Additionally, the 2015 survey was completed in late September, at the end of the warm season, while the 2011 survey was completed earlier in September. Still, these data indicate that heat warnings are reaching the majority of New Yorkers, even during a year that was not particularly severe for heat waves. Awareness of heat warnings was lower among lower-income respondents than higher-income respondents, which also differed from the 2011 survey, where people with incomes less than $30,000 were as likely to be aware of a warning as those with higher incomes [14]. 

Although heat warning penetration is relatively high, the perception that heat is a danger remains low among the general population. Less than half of New Yorkers reported a belief that on very hot days, the heat inside their home is very likely or somewhat likely to cause them to become ill. This matches findings of a North American multicity study, which found that most respondents were aware of heat warnings, but many did not consider themselves vulnerable to heat and few reported behavior changes to protect themselves [29]. These findings may partially be explained because a substantial fraction of the population has high adaptive capacity and is aware of this. Our results indicate that individuals from low-income households have higher risk perception than those from higher income households. These risk perceptions may simply accurately reflect the reality that low-income individuals perceive for themselves, in which they have limited resources to protect themselves from high heat exposure. 

The results of this study also provide insights into how New Yorkers perceive the risk of heat-health threats from climate change. According to the findings, two-thirds of the population was concerned about heat health-related effects of climate change. While some research has indicated that low-income individuals are disengaged from the problem of climate change [30], our results suggest that having a lower household income significantly predicts one’s perception of climate change as a health threat, reflecting an accurate perception of having fewer resources to adapt. These results echo findings in Maryland residents, where vulnerable populations (including those in low-income households) perceived their own climate change health vulnerability to be greater than the public at large [31]. Discrepancies between national and local surveys highlight the regional variation in climate change risk perception, which may be due to personal experience with extreme weather [17] among other factors, and underscores the importance of continued local research and preparedness efforts. Our findings suggest that low-income individuals may be particularly receptive to heat-health interventions and climate adaptation planning.

### 4.3. Heat-Protective Behaviors

In both 2011 and 2015, high proportions of residents reported staying home during very hot weather, even if they could not keep their home cool. In the 2015 survey we elicited more information about this behavior, finding that while low risk perception remained the most frequently reported reason for staying home, it was closely followed by a strong general personal preference (a volunteered response that was recorded, rather than a response option) and then by a specific preference not to spend time with strangers. These results point to the importance of (1) increasing access to home AC for those who are at risk and, (2) building a connected and resilient community prior to an emergency heat event to overcome the tendency toward social isolation. Social capital is a critical component of community resilience [32], and communities with robust social networks have had a greater ability to coordinate disaster recovery [33]. Social capital, or connectedness, can be increased through participatory decision-making, increased self-sufficiency, education of the public, and partnerships between government and nongovernmental organizations [34]. Our results on checking in on neighbors and family members indicate that low-income residents and racial minorities are already relying on social capital as a component of their resilience, which may be due to lack of access to other types of capital. These existing social networks can be leveraged in intervention design to improve the reach of information and the uptake of protective behaviors during heat events.

These results provide support to climate change adaptation initiatives focused on local preparedness, communication, and community engagement. Following the 2011 survey and focus groups conducted in 2012, the DOHMH reexamined and refined its risk communication strategies. That research pointed to a disconnect in risk perception, with vulnerable people recognizing that heat was dangerous in theory but not necessarily considering themselves to be at risk. DOHMH revised its summer heat brochure to further emphasize that heat could be deadly, highlight the indoor risk to vulnerable individuals without access to AC, and communicate the need to check on vulnerable individuals during extreme heat. To supplement the messaging campaign, DOHMH developed a postcard to provide guidance on how to check on vulnerable contacts. The agency developed “Talking Points” [35] guidance for those who communicate with the public about heat and worked with the local National Weather Service office to emphasize NYC-specific risks in the text of its heat products and to conduct outreach to local meteorologists about heat-health risks. DOHMH continues to refine its heat protection strategies using results from heat-health research and surveillance [8,9,36], including this study.

### 4.4. Limitations

While our survey captured valuable information that can be used by public health practitioners in NYC and beyond, several limitations should be noted, some of which also applied to the 2011 survey [14]. While every effort was made to achieve a high response rate in this study, including using a highly trained survey interview staff, callbacks on different days and at different hours, and attempted refusal conversions of soft refusals by trained refusal conversion specialists, we achieved a response rate of 22%. It should be noted that this is well above the current standard of approximately 9% in RDD telephone studies and there is no indication of nonresponse bias [37]. We used closed-ended survey questions, which may miss responses not included as options or overly simplify complex reasons for behaviors. Additionally, interpretation of questions may have varied by respondents. Due to funding constraints that limited the number of survey questions, we were unable to ask about residential building conditions that could affect participants’ ability to stay cool (other than AC ownership). It is now a necessity to use a dual-frame survey of landline and cell phone numbers for telephone surveys [38]; however, a 30% cell sample may underrepresent some segments of the population. Finally, although our survey sample size was sufficiently large to estimate prevalence, it may have been too small to test for statistical differences between subgroups in some cases. In particular, the size of the sample which chose to complete the survey in Spanish was small and we note that conducting a survey in only two languages in NYC may have limited participation. Future research should explore the relationship between language and heat vulnerability in more detail, as well as among more subsets of race and ethnicity categories.

## 5. Conclusions

Epidemiologic data have consistently demonstrated that people who have lower incomes, older adults, and black individuals are more vulnerable to extreme heat. This study demonstrates that non-Hispanic black individuals are more likely to lack access to AC, but this disparity is not fully explained by having a lower household income. This, along with the finding that people with low household incomes are more likely to lack access to AC, may partially explain reported disparities in heat-related morbidity and mortality. Further qualitative research is needed to investigate mechanisms for increased risk among non-Hispanic blacks, including other reasons for not owning AC beyond income constraints. As with the 2011 survey, our results underscore the need to continue to help vulnerable individuals purchase AC and run it during very hot weather. This could be done in part by expanding the cooling assistance component of the Home Energy Assistance Program, which currently receives less funding in New York state than the heating assistance component. AC access is a health-equity issue, one likely to become more urgent as our climate continues to warm. Our results also point to opportunities for knowledge building on the serious health consequences of heat and engagement with certain segments of the population on climate change adaptation. Periodic population-based surveys such as this provide insight into progress achieved and opportunities for advancement toward climate and health adaptation goals.

## Figures and Tables

**Table 1:** Characteristics of survey respondents (*n* = 801), New York City, 2015.

Category	Characteristic	Unweighted (N)	Weighted (%)
Sex	Male	311	47
Age	18–29	140	24
	30–49	233	37
	50–64	202	23
	65+	212	16
	Missing/Refused	14	
Race/ethnicity	White Non-Hispanic	246	34
	Black Non-Hispanic	189	20
	Hispanic	220	29
	Asian Non-Hispanic	59	12
	Other Non-Hispanic	37	5
	Missing/Refused	50	
Borough	Bronx	147	16
	Brooklyn	246	30
	Manhattan	148	21
	Queens	215	28
	Staten Island	45	6
Household Income	<$30,000	212	25
	$30,000–<$50,000	126	16
	$50,000–<$100,000	151	28
	≥$100,000	136	31
	Missing/Refused	176	
General Health Status	Excellent/Very Good/Good	668	88
	Fair/poor	125	12
	Missing/Refused	8	
AC ^1^ Status	No functioning AC	115	13
	Used never or < half time	120	15
	Used half the time or more	558	72
	Missing/refused	8	

^1^ AC: air conditioning.

**Table 2:** Reasons for not owning or not using AC ^1^, New York City, 2015.

Own/Use	Reason	Unweighted (N)	Weighted (%)	95% Confidence Interval
Does not own	Can’t afford it	44	40	(29.5, 49.6)
	Don’t need it	33	33	(22.8, 42.3)
	Don’t like AC	24	20	(12.4, 28.3)
	Building wiring not equipped	6	8	(1.5, 13.6)
Does not use ^2^	Electricity bill	52	24	(17.6, 29.7)
	Conserve electricity	47	21	(15.5, 27.3)
	Did not feel hot	38	17	(11.8, 23.0)
	Don’t like AC	27	12	(7.4, 16.6)
	Prefer fan	30	13	(8.0, 17.2)
	Go elsewhere	24	10	(5.8, 13.8)
	Health worse (volunteered)	8	3	(0.6, 5.8)

^1^ AC: air conditioning; ^2^ Reported using AC ‘never’ or ‘less than half the time’ when at home during very hot weather.

**Table 3:** Univariate and multivariate predictors of AC ^1^ access and use, New York City, 2015.

Outcome	Predictor	Univariate OR ^1^ (95% CI)	Univariate *p*-Value	Multivariate ^2^ OR (95% CI)	Multivariate ^2^ *p*-Value
Does not have AC	Income < $30 K	2.6 (1.6, 4.3)	<0.001	3.1 (1.8, 5.5)	<0.001
	Non-Hispanic black	1.9 (1.2, 3.1)	0.009	2.0 (1.1, 3.5)	0.028
	Male	1.0 (0.7, 1.5)	0.973	1.1 (0.6, 1.9)	0.760
	Age 65 and older	0.9 (0.5, 1.4)	0.528	0.5 (0.2, 1.1)	0.083
	Low risk perception	1.1 (0.7, 1.8)	0.584	1.6 (0.9, 2.9)	0.129
Does not use AC	Income < $30 K	1.2 (0.7, 2.0)	0.572	1.3 (0.7, 2.2)	0.373
	Non-Hispanic black	1.0 (0.6, 1.8)	0.905	1.2 (0.7, 2.3)	0.473
	Male	1.2 (0.8, 1.9)	0.326	1.3 (0.8, 2.2)	0.247
	Age 65 and older	1.1 (0.7, 1.8)	0.621	1.2 (0.7, 2.0)	0.608
	Low risk perception	1.4 (0.9, 2.2)	0.122	1.3 (0.8, 2.2)	0.353

^1^ OR: odds ratio, CI: confidence interval, AC: air conditioning; ^2^ Adjusted for all other factors listed in table.

**Table 4:** Univariate and multivariate predictors of heat awareness, heat risk perception, and climate change concern, New York City, 2015.

Outcome	Predictor	Univariate OR ^1^ (95% CI)	Univariate *p*-Value	Multivariate ^2^ OR (95% CI)	Multivariate ^2^ *p*-Value
Heat warning awareness	Non-Hispanic black	1.1 (0.7, 1.6)	0.740	0.9 (0.6, 1.4)	0.731
	Income < $30 K	0.7 (0.5, 1.0)	0.079	0.6 (0.4, 1.0)	0.034
	Male	0.8 (0.6, 1.1)	0.228	1.0 (0.7, 1.5)	0.889
	Age 65 and older	1.2 (0.8, 1.7)	0.369	1.2 (0.8, 1.9)	0.376
	Low risk perception	0.7 (0.5, 1.0)	0.075	0.6 (0.4, 0.9)	0.010
High risk perception ^3^	Non-Hispanic black	1.3 (0.9, 1.9)	0.179	1.2 (0.8, 1.8)	0.337
	Income < $30 K	1.8 (1.3, 2.6)	0.001	1.9 (1.3, 2.8)	0.001
	Male	0.8 (0.6, 1.1)	0.121	0.6 (0.4, 0.9)	0.010
	Age 65 and older	0.7 (0.5, 1.0)	0.060	0.7 (0.5, 1.1)	0.091
Concern ^4^	Non-Hispanic black	1.2 (0.8, 1.8)	0.298	1.3 (0.8, 2.1)	0.226
	Income < $30 K	1.5 (1.0, 2.2)	0.047	1.6 (1.0, 2.3)	0.032
	Male	0.9 (0.7, 1.3)	0.576	0.9 (0.6, 1.4)	0.786
	Age 65 and older	0.7 (0.5, 0.9)	0.023	0.7 (0.5, 1.0)	0.078

^1^ OR: odds ratio, CI: confidence interval; ^2^ Adjusted for all other factors listed in table; ^3^ Reported a belief that on very hot days, the heat inside their home is very likely or somewhat likely to make them ill; ^4^ Reported being “very concerned” or “somewhat concerned” that more frequent heat waves due to climate change would negatively impact their health.
